# Pharmacokinetics of Bismuth following Oral Administration of Wei Bi Mei in Healthy Chinese Volunteers

**DOI:** 10.1155/2020/2679034

**Published:** 2020-08-13

**Authors:** Lingye Zhang, Juan Liu, Fandong Meng, Yingying Guan, Yongjun Wang, Shengtao Zhu, Yaqiong Liu, Qing Xie, Junxian Yu, Shutian Zhang

**Affiliations:** ^1^Department of Gastroenterology, Beijing Friendship Hospital, Capital Medical University, National Clinical Research Center for Digestive Diseases, Beijing Digestive Disease Center, Beijing Key Laboratory for Precancerous Lesion of Digestive Diseases, Beijing, China; ^2^Department of Laboratorial Science and Technology, School of Public Health, Peking University, Beijing, China; ^3^Department of Pharmacy, Beijing Friendship Hospital, Capital Medical University, Beijing, China

## Abstract

**Background:**

Bismuth-containing quadruple therapy achieves higher eradication rate of *Helicobacter pylori*. High level of bismuth in blood may result in damage of many organs. Wei Bi Mei is a new bismuth-containing drug combining chemicals and Chinese medicine portions. The present research is to study the pharmacokinetics of bismuth to evaluate the safety and rational use of Wei Bi Mei granules. *Material and Methods*. Seven healthy Chinese adult subjects were enrolled in this research, which included a single-dose study and a multiple-dose study. Wei Bi Mei granules were administered orally to the subjects at corresponding time. Blood and urine were collected. All samples were analyzed by inductively coupled plasma mass spectrometry (ICP-MS).

**Results:**

For single-dose Wei Bi Mei granules administration, the mean time to peak concentration (*t*_max_) of bismuth was 2.29 ± 0.76 h, and the mean peak concentration (*C*_max_) of bismuth was 0.85 ± 0.55 ng/mL. For multiple-dose Wei Bi Mei granules administration, the *C*_max_ was 2.25 ± 1.18 ng/mL at day two, and the volume of distribution (*V*_*d*_) was (22.97 ± 9.82) × 10^3^ L. The urinary excretion of bismuth was the fastest during the first two days, with a mean excretion rate of 3.84 ± 1.23 ng/h. The bismuth concentration in urine was significantly reduced at day 16.

**Conclusion:**

Bismuth has a washout period of approximately two months. The concentration of bismuth in blood was far less than the “safe level.” Thus, Wei Bi Mei is a highly safe therapeutic medicine, with a good clinical application value. Wei Bi Mei should be recommended more widely for use in bismuth-containing quadruple therapy for the treatment of *Helicobacter pylori* infection.

## 1. Introduction

*Helicobacter pylori (H. pylori)* was first described by Warren and Marshall in 1984 [1, 2]. Research focusing on *H. pylori* infection has gained increasing interest in recent years due to its relationship with many diseases, including gastric cancer (GC), peptic ulcers, and gastric mucosa-associated lymphoid tissue lymphoma [3, 4]. Hence, eradication of *H. pylori* is widely recommended by many experts throughout the world [5, 6]. In China, the prevalence of *H. pylori* infection is 66% in rural areas and 47% in urban areas [7]. The high *H. pylori* infection rate is responsible for numerous diseases including GC, which is one of the most aggressive diseases with high incidence and mortality in China [8, 9]. For these reasons, eradication of *H. pylori* infection plays an important role in curing a serious of upper gastrointestinal tract diseases.

Recently, the global eradication rate of *H. pylori* has been decreasing [7, 10]. The rising antibiotic resistance rate to clarithromycin and metronidazole in the traditional triple therapy is the major cause of this decrease [8, 11]. Previous meta-analysis has shown that bismuth-containing quadruple therapy can overcome antibiotic resistance to some extent [12]. Additionally, the Toronto Consensus and Maastricht V/Florence Consensus Report have recommended bismuth-containing quadruple therapy (proton pump inhibitor + bismuth + two other antibiotics) as the first-line regimen for *H. pylori* eradication in areas with high drug resistance [6, 13].

Wei Bi Mei is a new drug combined chemicals and Chinese medicine portions, in which bismuth aluminate is a major ingredient. Different from common clinical drugs such as Bismuth Potassium Citrate Capsules and Colloidal Bismuth Pectin, Wei Bi Mei not only contains heavy magnesium carbonate and sodium bicarbonate, which can neutralize gastric acid, but also includes multiple Chinese herbal medicinal ingredients with special functions. These specific Chinese medicine portions include extract licorice, cortex frangulae, aloe, fructus foeniculi, and *Acorus gramineus*. Previous studies have shown the efficacy of Wei Bi Mei in *H. pylori* eradication and peptic ulcers treatment [14–16].

Nevertheless, bismuth mainly acts locally in the gut, and systemic absorption does not appear to be necessary for efficacy [17]. As a heavy metal element, high levels of bismuth may result in damage to the kidneys, bone joints, and central nervous system once entering into the human body [18–20].

There is no previous study focusing on the pharmacokinetics of bismuth in Wei Bi Mei in the human body. Hence, the aim of this study was to determine the pharmacokinetics of bismuth to evaluate the safety and rational use of Wei Bi Mei.

## 2. Materials and Methods

### 2.1. Materials, Reagents, and Test Formulation

The reference standards of bismuth (Bi, GSB04-1917-2004) and rhenium (Re, GSB04-1945-2004) were provided by Guobiao (Beijing) Testing & Certification Co., Ltd. Wei Bi Mei granules (20150406) were manufactured by Holwray Pharmaceutical (China) Co., Ltd. Ultra-pure grade water was produced by a Milli-Q water system (Millipore Inc., Bedford, MA, USA).

### 2.2. Instruments

Inductively coupled plasma mass spectrometry (ICP-MS) (DRC-II, Perkin Elmer Co., Ltd, USA) was used to determine bismuth levels under the following operating parameters. The nebulizer, auxiliary, and coolant gas flow rates were 1.0, 1.6, and 15.0 L/min, respectively. The dwell time was 100 ms, and the sample uptake flow rate was 1.0 mL/min. The sweep mode was set at single-point peak hopping, with a resolution ratio of 0.7–0.9. A microwave digestion system (UltraWAVE, Milestone Co., Ltd., Italy) was used under the following operation conditions: time, 5 min, 5 min, and 20 min; temperature, indoor, 150°C, 150°C, 190°C, 190°C, and 190°C, successively; forward power, 1300 W. A quartz digestion tube was used to dispense samples with 3 mL of nitric acid and 0.5 mL of hydrogen peroxide.

### 2.3. Sample Pretreatment

The analysis of bismuth was performed by ICP-MS. An aliquot of 1.0 mL of plasma was mixed with 3 mL of HNO_3_ and 0.5 mL of H_2_O_2_ and solubilized by microwave digestion. After cooling the mixture, distilled water was added to get a final volume of 8 mL. Four times of dilution were done until the final concentration of internal standard (IS) rhenium became 0.1 ng/mL. An aliquot of 0.4 mL of urine sample was mixed with HNO_3_ (1%) and IS solution to make a volume of 10 mL and the final concentration of IS was 0.5 ng/mL.

### 2.4. Subjects and Procedures

The clinical trial protocol was approved by the Institutional Review Board of Beijing Friendship Hospital (no. BJFH-EC/2014-012) and registered online in the Chinese Clinical Trial Registry (no. ChiCTR-ONC-14004656). The informed consent was obtained from all participants prior to the study.

Seven healthy Chinese adult subjects were enrolled in this study. The mean age of the participants was 25.4 ± 2.2 years (range 23–28 years) and the mean body mass index height was 22.1 ± 2.2 kg/m^2^ (range 17.6–23.6 kg/m^2^). All physical signs were normal. The participants were nonsmokers and did not recently use medications. The use of tobacco, caffeine, or any medications was also prohibited during the study. Clinical parameters were also monitored to evaluate the health of these subjects during the trial, which mainly included alanine aminotransferase activity, alkaline phosphatase activity, total protein, albumin, total bilirubin, direct bilirubin, glucose, creatinine, blood urea nitrogen, aspartate aminotransferase activity, potassium ions, sodium ions, and chloride ions.

The subjects were admitted to the hospital clinical research ward in the morning after fasting from solids and liquids which was started at 10 : 00 pm the previous night. The fasting blood samples and urine samples of the subjects were collected early in the morning of the day of testing.

For single-dose Wei Bi Mei granules test, subjects orally administer one bag of Wei Bi Mei granules (containing 200 mg of bismuth) at 30 min after breakfast on day one. Blood Samples (brachial vein blood) were collected at 10 min, 30 min, 60 min, 90 min, 2 h, 3 h, 4 h, 6 h, 8 h, 12 h, and 24 h after ingestion. Then, the whole blood was centrifuged, and the plasma was stored in the freezer at −70°C until analysis.

**Figure 1:**
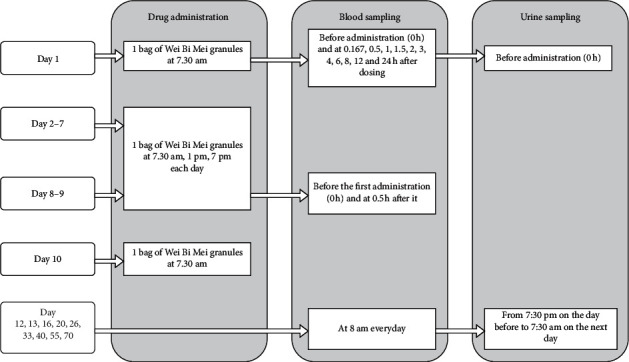
Flow chart of the study.

For multiple-dose Wei Bi Mei granules test, subjects were orally administered one bag of Wei Bi Mei granules after meal three times a day (at 7 : 30 am, 1 : 00 pm, and 6 : 30 pm) on days two to nine. Blood samples were collected on day eight and day nine at 30 min before and 30 min after the first daily administration. The drug was stopped on day 10 after the oral administration of one bag of Wei Bi Mei granules. Then, the blood samples were collected on days 12, 13, 16, 20, 26, 33, 40, 55, and 70. Urine samples were collected on days 12, 13, 16, 20, 26, 33, 40, 55, and 70 over a period of 12 h (from voiding bladder at 7 : 30 pm on the day before to 7 : 30 am on the next day) to record the volume after blending. All samples were stored in the freezer at −70°C until analysis. The flow chart of this study is shown in Figure 1.

### 2.5. Pharmacokinetics Study

The pharmacokinetics of Wei Bi Mei were evaluated using DAS 2.2 (Mathematical Pharmacology Professional Committee of China, Shanghai, China). Pharmacokinetic analysis included determination of the following parameters: maximum concentration (*C*_max_); time of maximum concentration (*t*_max_); apparent elimination rate constant (Kel); AUC, the area under the plasma concentration-time curve (AUC_0-*t*_), calculated by the linear trapezoidal rule; mean residence time (MRT); *V*_*d*_, volume of distribution; elimination half-life (*t*_1/2_).

## 3. Results

### 3.1. Method Validation

The lower limit of quantification (LLOQ) and limit of detection (LOD) were 0.4 ng/mL and 0.04 ng/mL for bismuth in plasma samples. LLOQ and LOD were 0.1 ng/mL and 0.02 ng/mL for bismuth in urine samples. The method was linear for bismuth from 0.04 to 5 ng/mL in plasma (*R*^2^ = 0.9997) and 0.2–10 ng/mL in urine (*R*^2^ = 0.9998). The mean recoveries of bismuth from plasma and urine were 96.26 ± 1.18% and 89.84 ± 1.14%, respectively. The intra- and interday coefficients of variation and percentage error values of the assay method were all less than 10%.

### 3.2. Pharmacokinetics Study

**Figure 2:**
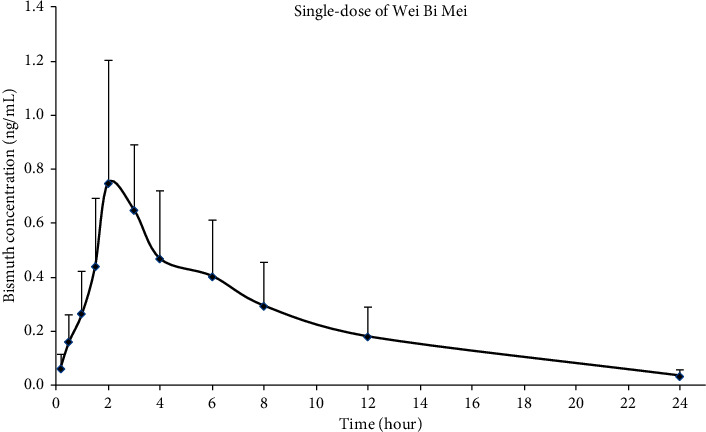
Mean plasma concentration-time profile of bismuth following a single-dose Wei Bi Mei granules administration (mean ± SD, *n* = 7).

For single-dose pharmacokinetics of bismuth, the median bismuth plasma concentration–time curves are shown in Figure 2, and the pharmacokinetic parameters of bismuth are listed in Table 1. The mean *C*_max_ was 0.85 ± 0.55 ng/mL occurring at 2.29 ± 0.76 h. The *t*_1/2_ of bismuth was 9.98 ± 5.13 h and the mean value of AUC_0–24_ was 8.33 ± 5.42 h ng/mL.

**Figure 3:**
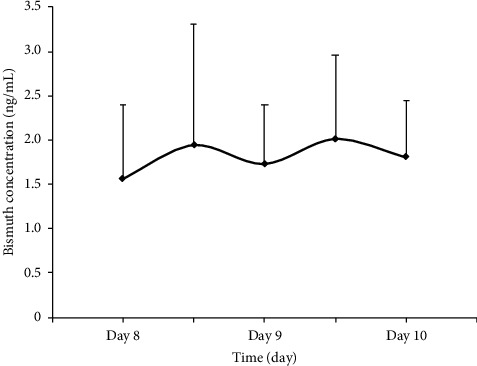
Mean plasma concentration-time profile of bismuth in the steady state (on days eight to ten) following multiple-dose Wei Bi Mei granules administration (mean ± SD, *n* = 7).

**Figure 4:**
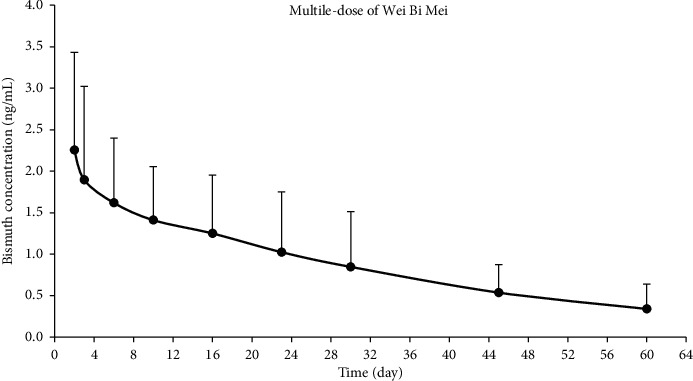
Mean plasma concentration-time profile of bismuth after multiple-dose Wei Bi Mei granules administration (mean ± SD, *n* = 7).

For multiple-dose pharmacokinetics of bismuth, seven subjects were orally administered Wei Bi Mei granules three times a day on days two to nine. As shown in Figure 3, the trough concentrations (1.55 ± 0.84 ng/mL and 1.73 ± 0.67 ng/mL at 30 min before administration, resp.) were similar to the peak concentrations (1.94 ± 1.37 ng/mL and 2.01 ± 0.95 ng/mL at 30 min after administration, resp.), which means the bismuth concentration reached a steady state between day eight and day nine.  Figure 4 shows the plasma concentration-time curve after withdrawal of Wei Bi Mei granules. The pharmacokinetic parameters of bismuth are listed in Table 2. The *t*_max_ occurred on day two. Noteworthy, the *C*_max_ was 2.25 ± 1.18 ng/mL, which was significantly greater than that of the single-dose administration on day one (0.85 ± 0.55 ng/mL). The volume of distribution (*V*_*d*_) was (22.97 ± 9.82) × 10^3^ L, which was far greater than the fluid volume of the whole body (approx. 50 L). This finding suggested that bismuth easily bound with tissues in the body, resulting in accumulation. The mean residence time (MRT) of bismuth in the whole blood was 21.69 ± 3.95 days. In addition, the plasma concentration–time curve showed that participants still had residual bismuth in tissues and organs at 60 days after drug withdrawal.

### 3.3. Urinary Excretion of Bismuth

**Figure 5:**
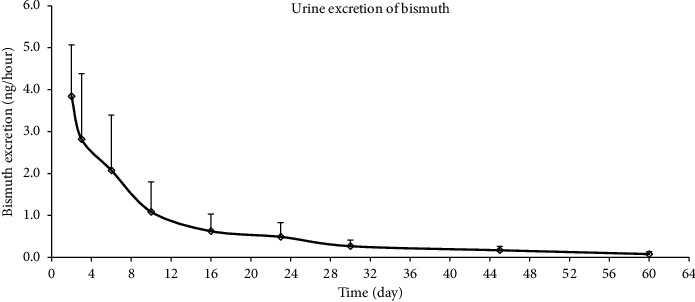
Mean urinary excretion profile of bismuth following drug withdrawal after multiple-dose Wei Bi Mei granules administration (mean ± SD, *n* = 7).

Bismuth urinary excretion was investigated for up to 60 days after the last dose. The concentration-time curve of bismuth in urine is shown in Figure 5; each time point represents the urinary excretion of bismuth over a 12 h interval. From the graph, the bismuth excretion could be divided into an early fast excretion phase and a later slow elimination phase. In the fast excretion phase, the bismuth excretion rate was the fastest during the first two days with an average excretion rate of 3.84 ± 1.23 ng/h. The bismuth concentration in the urine was significantly reduced on day 16, with a mean excretion rate of 0.62 ng/h. The bismuth excretion curve tended to be stable in the slow elimination phase two weeks after drug withdrawal, and the mean excretion rate was 0.08 ± 0.05 ng/h on day 60.

### 3.4. Safety and Tolerability

Seven subjects completed the study as planned. One subject withdrew from participation due to diarrhea on the second day of the oral administration of Wei Bi Mei granules and had dark green watery stools about three to four times a day, without abdominal pain and tenesmus. The diarrhea stopped on the second day after the withdrawal of Wei Bi Mei granules. No similar or other adverse events appeared in the remaining subjects. All clinical parameters tested with the blood samples of subjects during the experiments remained within normal ranges.

## 4. Discussion

Wei Bi Mei is a new bismuth-containing drug with heavy magnesium carbonate, sodium bicarbonate, and multiple Chinese herbal medicinal ingredients to treat *H. pylori* infection. As a heavy metal element, bismuth has the potential to damage human organs, such as the kidneys, bone joints, and central nervous system [18–20]. Thus, it is vital to evaluate the safety of Wei Bi Mei by studying the pharmacokinetics of bismuth in human body. In the present study, we found that Wei Bi Mei is safe in clinical application for its low concentration of bismuth in human blood. Based on this result, we recommend Wei Bi Mei to be used in bismuth-containing quadruple therapy for *H. pylori* eradication.

To the best of our knowledge, this is the first study to detect bismuth levels in human samples treated with Wei Bi Mei by ICP-MS. The developed method was highly sensitive for detection of bismuth and solved the problem that the level of bismuth was too low in human specimens to be detected by general methods.

Hillemand et al. reported that the “safe level” concentration of bismuth is 50–100 ng/mL in blood (80–160 ng/mL in plasma) and the “alarming level” concentration is over 100 ng/mL in blood (>160 ng/mL in plasma) [21]. In this study, the *C*_max_ of bismuth in whole blood was 0.85 ± 0.55 ng/mL after a single-dose oral administration of Wei Bi Mei granules and *C*_max_ of bismuth in the blood was 2.25 ± 1.18 ng/mL after the multiple-dose oral administration of Wei Bi Mei granules on day 10. Besides, the mean steady state concentration was 1.81 ng/mL, which was far less than the “safe level.” Based on these results, Wei Bi Mei has a high safety profile and a low probability of bismuth-related toxic reactions.

In the present study, bismuth is excreted slowly in plasma and even more slowly in urine. Most of the bismuth could be eliminated from the body within 60 days which indicated that consecutive courses of treatment should be paused after this time period in clinical practice. In addition, Zhang et al. indicated that bismuth might be reabsorbed by kidney tubules and accumulate in kidney [22]. Li et al. showed that bismuth was absorbed and retained mainly in the kidneys and Wei Bi Mei yielded the lowest accumulation of bismuth in the kidneys compared with colloidal bismuth subcitrate and bismuth aluminate [14]. Hence, kidneys may be the primary organs of bismuth accumulation and Wei Bi Mei is safer than other bismuth compounds.

In conclusion, pharmacokinetic analyses indicated that bismuth has a washout period of approximately two months. Besides, the concentration of bismuth in blood was far less than the “safe level” and thus Wei Bi Mei is safe in clinical practice. Wei Bi Mei could be recommended for wide use in bismuth-containing quadruple therapy.

## Figures and Tables

**Table 1:** Pharmacokinetic parameters of bismuth after a single oral dose of Wei Bi Mei (mean ± SD, *n* = 7).

Parameter	*C*_max_ (ng/mL)	*t*_max_ (h)	*t*_1/2_ (h)	*V*_*d*_ (×10^3^ L)	AUC_0–24_ (h ng/mL)	AUC_0-∞_ (h ng/mL)	MRT (0-*t*) (h)
	0.85 ± 0.55	2.29 ± 0.76	9.98 ± 5.13	196.47 ± 98.79	8.33 ± 5.42	8.43 ± 5.08	11.82 ± 6.78

*C*_max_, peak concentration; *t*_max_, time to peak concentration; *t*_1/2_, half-life; *V*_*d*_, volume of distribution; AUC, area under the curve; MRT, mean residence time.

**Table 2:** Pharmacokinetic parameters of bismuth following multiple-dose Wei Bi Mei administration (mean ± SD, *n* = 7).

Parameter	*C*_max_ (ng/mL)	*t*_max_ (d)	*t*_1/2_ (d)	*V*_*d*_ (×10^3^ L)	AUC_0-t_ (d ng/mL)	AUC_0-∞_ (d ng/mL)	MRT (0-*t*) (d)
	2.25 ± 1.18	2.0	20.04 ± 10.65	22.97 ± 9.82	55.26 ± 32.01	58.68 ± 36.64	21.69 ± 3.95

*C*_max_, peak concentration; *t*_max_, time to peak concentration; *t*_1/2_, half-life; *V*_*d*_, volume of distribution; AUC, area under the curve; MRT, mean residence time.

## Data Availability

All data are present within the text.

## References

[1] Marshall B., Warren J. R. (1984). Unidentified curved bacilli in the stomach of patients with gastritis and peptic ulceration. *The Lancet*.

[2] Warren J. R., Marshall B (1983). Unidentified curved bacilli on gastric epithelium in active chronic gastritis. *Lancet*.

[3] Bjorkman D. J., Steenblik M. (2017). Best practice recommendations for diagnosis and management of *Helicobacter pylori*-synthesizing the guidelines. *Current Treatment Options in Gastroenterology*.

[4] Chey W. D., Leontiadis G. I., Howden C. W., Moss S. F. (2017). ACG clinical guideline: treatment of *Helicobacter pylori* infection. *American Journal of Gastroenterology*.

[5] Sugano K., Tack J., Kuipers E. J. (2015). Kyoto global consensus report on *Helicobacter pylori* gastritis. *Gut*.

[6] Fallone C. A., Chiba N., van Zanten S. V. (2016). The Toronto consensus for the treatment of *Helicobacter pylori* infection in adults. *Gastroenterology*.

[7] Nagy P., Johansson S., Molloy-Bland M. (2016). Systematic review of time trends in the prevalence of *Helicobacter pylori* infection in China and the USA. *Gut Pathogens*.

[8] Flores-Treviño S., Mendoza-Olazarán S., Bocanegra-Ibarias P., Maldonado-Garza H. J., Garza-González E. (2018). *Helicobacter pylori* drug resistance: therapy changes and challenges. *Expert Review of Gastroenterology & Hepatology*.

[9] Sun K. X., Zheng R. S., Zhang S. W. (2015). Report of cancer incidence and mortality in different areas of China. *China Cancer*.

[10] Burucoa C., Axon A. (2017). Epidemiology of *Helicobacter pylori* infection. *Helicobacter*.

[11] Fallone C. A., Moss S. F., Malfertheiner P. (2019). Reconciliation of recent *Helicobacter pylori* treatment guidelines in a time of increasing resistance to antibiotics. *Gastroenterology*.

[12] Venerito M., Krieger T., Ecker T., Leandro G., Malfertheiner P. (2013). Meta-analysis of bismuth quadruple therapy versus clarithromycin triple therapy for empiric primary treatment of *helicobacter pylori* infection. *Digestion*.

[13] Malfertheiner P., Megraud F., O’Morain C. A (2017). Management of *Helicobacter pylori* infection-the Maastricht V/Florence consensus report. *Gut*.

[14] Li L., Meng F., Zhu S. (2018). Efficacy and safety of Wei Bi Mei, a Chinese herb compound, as an alternative to bismuth for eradication of *Helicobacter pylori*. *Evidence-Based Complementary and Alternative Medicine*.

[15] Li Q., Wang N., Hu F., Li J., Yang G. (2016). Study of compound bismuth and magnesium granules on clearance of *helicobactor pylori* infection in KM mice. *International Journal of Clinical and Experimental Medicine*.

[16] Wang H., Yu B., Dong Q. (2017). Effect of compound bismuth and magnesium granules quadruple therapy on Hp-eradication. *China Mod Medicine*.

[17] Gavey C. J., Szeto M. L., Nwokolo C. U., Sercombe J., Pounder R. E. (1989). Bismuth accumulates in the body during treatment with tripotassium dicitrato bismuthate. *Alimentary Pharmacology & Therapeutics*.

[18] Eichler I. (1979). The bismuth-encephalopathy-a new pathological syndrome (author’s transl). *Wiener Klinishce Wochenschr*.

[19] Borbinha C., Serrazina F., Salavisa M., Viana-Baptista M. (2019). Bismuth encephalopathy-a rare complication of long-standing use of bismuth subsalicylate. *BMC Neurology*.

[20] Larsen A., Stoltenberg M., West M. J., Danscher G. (2005). Influence of bismuth on the number of neurons in cerebellum and hippocampus of normal and hypoxia-exposed mouse brain: a stereological study. *Journal of Applied Toxicology*.

[21] Hillemand P., Pallière M., Laquais B., Bouvet P. (1977). Bismuth treatment and blood bismuth levels. *Sem Hop*.

[22] Zhang S. T., Zhang S. H., Yu Z. L., Wang Y. F. (2002). The difference of the bismuth absorption from a single colloidal bismuth pectin therapy and quadruple therapy for eradicating *Halicobactor pylori* infection. *National Medical Journal of China*.

